# Analysis of the Impact of the Presence of Phylum Cyanobacteria in the Microbiome of Patients with Breast Cancer on Their Prognosis

**DOI:** 10.3390/jcm11247272

**Published:** 2022-12-07

**Authors:** Jeongshin An, Bum-Jun Kil, Hyungju Kwon, Young Ju Kim

**Affiliations:** 1 Institute of Convergence Medicine Research, Ewha Womans University Mokdong Hospital, College of Medicine, Ewha Womans University, 1071 Anyangcheon-ro, Yangcheon-gu, Seoul 07985, Republic of Korea; 2 Department of Surgery, Ewha Womans University Mokdong Hospital, College of Medicine, Ewha Womans University, 1071 Anyangcheon-ro, Yangcheon-gu, Seoul 07985, Republic of Korea; 3 Department of Maritime Operation, ROK Naval War College, Jaun-ro, Yuseong-gu, Daejeon 34059, Republic of Korea; 4 Department of Obstetrics and Gynecology, Ewha Medical Institute and College of Medicine, Ewha Womans University, Seoul 07804, Republic of Korea

**Keywords:** Cyanobacteria, breast cancer, microbiome, prognostic factor

## Abstract

Cyanobacterial blooms caused by Cyanobacteria adversely affect the health of the people living in their vicinity. We elucidated the effect of Cyanobacteria in patients with breast cancer. The serum microbiome of the patients with breast cancer was analyzed using NGS. Serologic tests were performed to analyze the association between the factors affecting the liver function of patients with breast cancer and the amount of Cyanobacteria. In addition, the recurrent-free survival of patients with breast cancer according to the abundance of Cyanobacteria was analyzed. The abundance of Cyanobacteria tended to be correlated with the serological results related to liver function. A high abundance of Cyanobacteria seemed to be more related to late-stage breast cancer. A high recurrent-free survival was related to a low abundance of Cyanobacteria. Even though no toxicity study was conducted, this study demonstrates the impact of phylum Cyanobacteria on the prognosis of patients with breast cancer. Thus, the abundance of Cyanobacteria in the microbiome can help predict the prognosis of patients with breast cancer.

## 1. Introduction

Breast cancer is the most common cancer among women worldwide, and its incidence is still increasing [1]. In addition, its recurrence rate is high. For example, triple-negative breast cancer is known to have a high recurrence rate within five years and a poor prognosis [2]. Moreover, luminal-type breast cancer may recur even after 5 or 10 years, when the patient thinks that the disease has already been overcome [3]. Therefore, the prognosis and management of breast cancer are important. The importance of eating habits, which are the primary breast cancer prevention strategy, is well known [4]. The diet and lifestyle, along with the environment in which the patient lives, play an important role in preventing breast cancer, according to this study. We present evidence that supports the impact of the environment on breast cancer development. Genetic problems cannot be solved; however, various risk factors, such as the environment, can be improved. In this study, we investigate the presence of Cyanobacteria in the microbiome of patients with breast cancer, which reflects the environment that surrounds them and can affect their prognosis.

The elements derived from symbiotic microbiota found in the human microbiome include the DNA of bacteria, fungi, and viruses, as well as archaea [5]. Cyanobacteria were formerly called blue-green algae and classified as eukaryotes, but they are now classified as prokaryotes [6]. This study focused on the sequence of the phylum Cyanobacteria in the human microbiome rather than the bacteria themselves. Cyanobacteria can also be found in the human microbiome [7,8] and are often found in stagnant or polluted water, such as in reservoirs or lakes [9]. The presence of Cyanobacteria in freshwater is associated with suspended cyanobacterial blooms and toxins [10]. In particular, with global warming acceleration, the water temperature is rising. Eutrophication due to the presence of domestic sewage and a high water temperature creates the optimal conditions for the growth of Cyanobacteria [11]. The accelerating growth of Cyanobacteria in freshwater worldwide has been studied using the Landsat 5 satellite data from the National Aeronautics and Space Administration (NASA) [12]. In this study, cyanobacterial blooms in Republic of Korea were identified using data from the Sentinel-2 satellite of the European Space Agency. Cyanobacteria can not only be absorbed into the human body by directly drinking contaminated water but also through recreational activities [13]. In addition, according to previous studies, the consumption of fish and shellfish and vegetables grown with contaminated water can affect the concentration of Cyanobacteria in the human body [14,15].

Cyanobacteria produce various toxins that can cause problems in the liver, nervous system, and genes. Examples are microcystin, nodularin, anatoxin, saxitoxin, and cylindrospermopsin. [14]. The most commonly found cyanobacterial toxins are microcystins and anatoxins [14]. While acute exposure to microcystins causes nausea, vomiting, abdominal pain, and skin rash [16], chronic exposure to this toxin can cause malignancy, such as colon or liver cancer [14]. In particular, microcystin-LR can act as a tumor promoter in human cancer development [17].

The relationship between breast cancer and Cyanobacteria has not been studied. Since Cyanobacteria are associated with the development of certain malignancies [15], it is important to study their effects on breast cancer. The presence of cyanobacterial DNA in the human microbiome may not be a result of short-term exposure to Cyanobacteria. It results from cumulative exposure to Cyanobacteria toxins since birth through the food chain [18]. The effect of long-term exposure to Cyanobacteria on the prognosis of patients with breast cancer was analyzed by testing the blood microbiome of patients. In particular, here, we focused on the presence of bacterial extracellular vesicles in the blood of patients. Bacterial extracellular vesicles contain nucleic acids and metabolites and are present in all body fluids, especially in the blood [19]. Previous studies have shown that there is a relationship between the composition of the microbiome in extracellular vesicles of body fluids and breast cancer [20,21]. In this study, the effect of a phylum Cyanobacteria-containing microbiome on the prognosis of patients with breast cancer was investigated. Our results will help explain the effect of a Cyanobacteria-polluted environment, which is reflected in the microbiome of breast cancer patients, on their prognosis.

## 2. Materials and Methods

### 2.1. Recruitment of Patients

Female patients diagnosed with breast cancer and presenting pathologic results were recruited. After the pathologic diagnosis of breast cancer, their sera were collected before treatment including surgery, chemotherapy, or radiation therapy. The subjects had no history of taking drugs or supplements such as antibiotics or probiotics within one month before blood sampling. Informed consent was obtained from the study participants. The research was conducted with the approval of the respective institutional review board (the approval number: Ewha Womans University Mokdong Hospital, EUMC 2014-10-005-019). At the time of initial recruitment, the patients with stage 0–III breast cancer with no metastasis or recurrence were recruited and were followed up for 7 or 8 years.

### 2.2. Distribution of Cyanobacteria Blooms in Korean Freshwater according to the Satellite

The Copernicus Sentinel-2 satellite, which has a high-resolution multispectral sensor, was used to analyze the status of cyanobacterial blooms in the riverine area of the Korean peninsula. The Ulyssys Water Quality Viewer (UWQV), whose information derives from Sentinel-2, was utilized to identify the cyanobacterial blooms, assuming that these can be identified by the colored UWQV values [22]. The Sentinel Hub EO Browser was implemented for analysis of the imagery. For data analysis, the highly concentrated UWQV value areas were compared with the low-UWQV-value areas from July to August 2022.

### 2.3. Isolation of the Extracellular Vesicles and DNA Extraction

Serum samples were collected from the participants using the same method as that used in the previous studies [20,21]. Blood samples were collected into serum vacuum separator tubes. For each sample, the serum was isolated from the blood, and then the extracellular vesicles (EVs) were collected. To isolate the EVs, the serum was centrifuged at 1500× *g* for 15 min at 4 °C and diluted in 1× phosphate-buffered saline (PBS, pH 7.4, ML008-01; Welgene, Gyeongsan, Republic of Korea). Thereafter, centrifugation was performed at 10,000× *g* at 4 °C for 1 min. Then, the supernatant was filtered using a 0.22 μm filter and ultracentrifuged at 150,000× *g* for 3 h at 4 °C using a 45 Ti rotor (Beckman Instruments, Brea, CA, USA). The obtained pellet, containing the EVs, was diluted in PBS and stored at −80 °C. Thereafter, an isolation kit (MoBio PowerSoil DNA Isolation Kit; Qiagen, Hilden, Germany) was used to obtain the DNA. DNA was extracted from the EVs according to the manufacturer’s instructions. The extracted DNA was quantified using a QIAxpert system (Qiagen).

### 2.4. Next-Generation Sequencing for Microbiome Analysis

Next-generation sequencing was performed based on the EV extraction results of the participants and on the bacteria-specific 16s rDNA—in particular, the V3–V4 region, which is a hypervariable region. For this, the MiSeq system and application (Illumina, San Diego, CA, USA) suitable for the NGS of bacteria were used. The primers used and the corresponding sequences are the following: 16S_V3_F (5′-TCGTCGGCAGCGTCAGATGTGTATAAGAGACAGCCTACGGGNGGCWGCAG-3′) and 16S_V4_R (5′-GTCTCGTGGGCTCGGAGATGTATAAGAGAGACAGGAC-TACHVGGGTATCTAATCC-3′), which are the same as those used in the previous studies [20,21]. Libraries were prepared, and each amplicon was sequenced according to the manufacturer’s guidelines. MDx-Pro ver.1 (MD Healthcare, Seoul, Republic of Korea), a profiling program, was used for the taxonomic assignment. The read length was set as 300 bp, and the average quality score was set to >20. The operational classification units were based on the CD-HIT cluster database, and the UCLUST algorithm was used to divide a set of sequences into clusters. Moreover, QIIME, a microbiome bioinformatics platform, and Greengenes were used to analyze the microbial communities. The results were analyzed from the phylum to the species level. In particular, the ratio of the Cyanobacteria phylum to other phyla was analyzed, and the microbiome with the highest Cyanobacteria ratio at the species level was identified. Among the microbiomes, the microbiome assigned to ‘*Uncultured Cyanobacterium*’ was the most studied.

### 2.5. Analysis of the Percentage of Cyanobacteria in the Microbiome of Patients with Breast Cancer

The microbiomes of patients with breast cancer were analyzed in terms of alpha and beta diversity (Figure S1). The percentage of Cyanobacteria was compared with that of other phyla using dot plot graphs. The relationship between the ratio of Cyanobacteria and the factors affecting breast cancer was analyzed. The accumulation of Cyanobacteria according to time was analyzed by investigating the relationship between the absence or presence of phylum Cyanobacteria and the age of patients. The proportion of phylum Cyanobacteria in women undergoing menopause, which is a factor that affects hormone levels, was investigated. The relationship between the percentage of phylum Cyanobacteria and the stage, tumor size, and lymph node metastasis was analyzed. The association between the ratio of phylum Cyanobacteria and factors related to liver metabolism was investigated. Serological test results, such as the low-density lipoprotein (LDL) cholesterol, high-density lipoprotein (HDL) cholesterol, and fasting blood glucose levels, were obtained. Since most of the participants did not have AST (aspartate aminotransferase) and ALT (alanine transaminase) levels higher than three times the normal value (>120 IU/L), which are the liver disease criteria [23], no patient was diagnosed with liver disease. Therefore, the ALP (Alkaline phosphatase) level was used to analyze the relevance of Cyanobacteria to liver function. The interaction between the ratio of phylum Cyanobacteria and the bacteria affecting liver function, such as the genus *Lactobacillus* and the family *Ruminococcaceae*, was investigated [24,25]. Finally, Kaplan–Meier was used to investigate the metastasis and recurrence of patients with breast cancer after a certain time elapsed, according to the abundance of phylum Cyanobacteria.

## 3. Results

### 3.1. Characteristics of Patients

For microbiome analysis, 96 patients with breast cancer participated in this study, which was conducted as part of another large project. The microbiome was analyzed through serological testing prior to the treatment of patients with breast cancer. The average age of the patients was 51 years. Body mass index (BMI) was classified into four groups according to the World Health Organization (WHO) classification: underweight (<18.5 kg/m^2^), normal weight (18.5–24.9 kg/m^2^), overweight (25–29.9 kg/m^2^), and obesity class I (30.0–34.9 kg/m^2^) [26]. The menstruation and eating habit characteristics of the patients with breast cancer were studied through questionnaires. The postoperative biopsy results were used to analyze the breast cancer stages, the subtypes, the tumor size, and the number of metastatic lymph nodes. For the serological testing of the patients, the high-density lipoprotein (HDL) and low-density lipoprotein (LDL) cholesterol, alkaline phosphatase (ALP), and fasting glucose levels were measured. There were no patients with diabetes mellitus among the recruited subjects, and there was no case of a fasting blood glucose of 126 mg/dL or higher. None of the patients were smokers or alcoholics (Table 1).

### 3.2. Analysis of the Cyanobacterial Blooms in Republic of Korea

**Figure 1:**
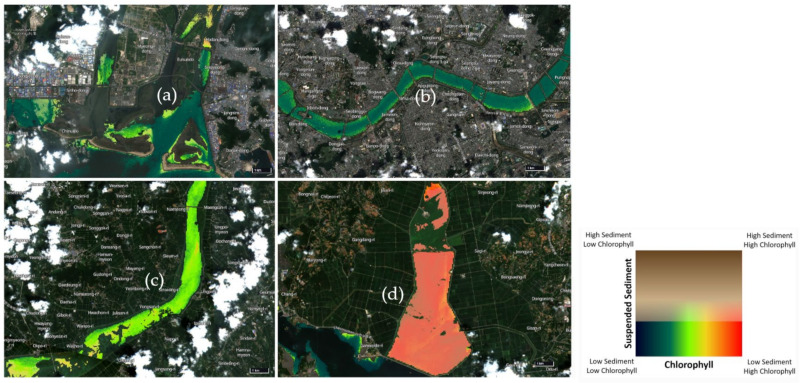
Analysis of the cyanobacterial blooms in Republic of Korea, according to the Sentinel-2 satellite data. (**a**) Data showing the cyanobacterial blooms near Eulsukdo, in the Nakdong River estuary, (**b**) in the Han River, (**c**) in the Geum River estuary, (**d**) and in the Ganwol Lake, an artificial lake in Cheonsu Bay. The color indicates the level of cyanobacterial blooms; the red color shows the most severe cyanobacterial blooms, and the black color shows that there are no cyanobacterial blooms in the freshwater.

As shown in Figure 1, from July to August 2022, abnormally high UWQV values were observed in the Ganwol Lake, which is an artificial lake in Cheonsu Bay. The Nakdong and Geum Rivers also showed high UWQV values, unlike the Han River. The Ganwol Lake is likely polluted by industrial factors. Conversely, the Han River shows some cyanobacterial blooms that are not as severe as those of the Nak-dong and Geum Rivers.

### 3.3. Characteristics of the Cyanobacteria Phylum

**Figure 2:**
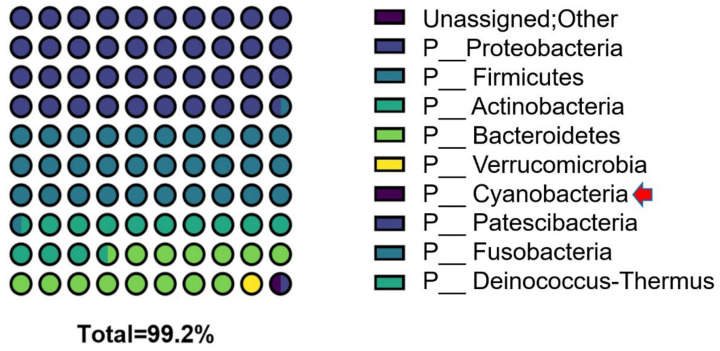
A 10 × 10 dot plot graph resulting from the analysis of the microbiome of patients with breast cancer at the phyla level, showing the relative proportion of phylum Cyanobacteria (red arrow) in their microbiome. P: phylum.

The average percentage of phylum Cyanobacteria in the human microbiome is less than 1%. Figure 2 shows the relative proportion of phylum Cyanobacteria to other phyla in patients with breast cancer. The proportion of the top 10 phyla in the patients with breast cancer was 99.2%, and the most common phylum in these patients was Proteobacteria, followed by Firmicutes and Bacteroidetes. The average percentage of Cyanobacteria sequences in the patients was 0.45%, which was lower than that of Verrucomicrobia sequences but similar to that of Patescibacteria sequences (Figure 2).

**Figure 3:**
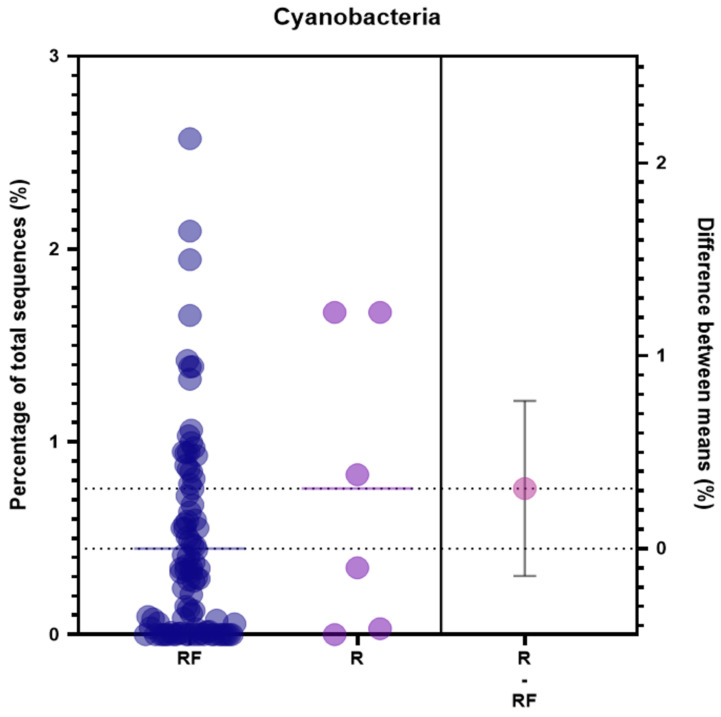
The level of the phylum Cyanobacteria in each group of patients with breast cancer. The relative proportion of phylum Cyanobacteria in patients with recurrence-free and recurrence states was compared. RF: patients without recurrence during more than 6 years of follow-up; R: patients with recurrence during more than 6 years of follow-up; RF-R: mean difference in phylum Cyanobacteria between the two patient groups.

The patients with breast cancer participating in our study were followed up for 7–8 years, and the rate of recurrence was analyzed. The patients with breast cancer were divided into two groups: patients who were recurrence-free and those with recurrence. The amount of phylum Cyanobacteria in the microbiome of the patients of the two groups was compared. The average amount of Cyanobacteria in the microbiome was higher in recurrent patients than it was in patients who were recurrence-free (Figure 3). The urine microbiome was further analyzed to compare it with the serum microbiome data from breast cancer patients. As a result, there was no statistically significant difference in the number of the sequences of Cyanobacteria in serum and urine (Figure S3).

**Figure 4:**
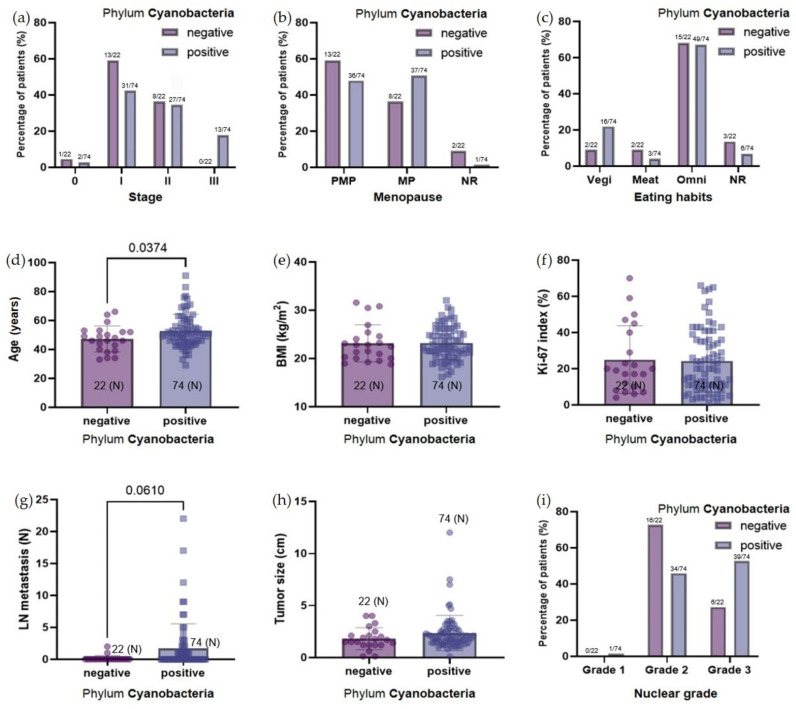
Bar graphs showing the relationship between the presence or absence of Cyanobacteria in the microbiome and the characteristics of the patients with breast cancer. (**a**) Percentage of patients according to cancer stage. (**b**) Percentage of patients according to menopause or not. (**c**) Percentage of patients according to eating habits. (**d**) The average age according to the presence or absence of Cyanobacteria. (**e**) The average BMI according to the presence or absence of Cyanobacteria. (**f**) The average Ki67 index according to the presence or absence of Cyanobacteria. (**g**) The number of lymph node metastases according to the presence or absence of Cyanobacteria. (**h**) Tumor size according to the presence or absence of Cyanobacteria. (**i**) Nuclear grade according to the presence or absence of Cyanobacteria. PMP: premenopausal patients; MP: patient with menopause; NR: nonresponse; Vegetarian: patients who eat only vegetarian food; Meat-based diet: patients who eat meat-based foods daily; Omnivorous: patients who eat any food well; NR: nonresponse; BMI: body mass index; LN: lymph node; (N): number of patients.

The influence of the presence of phylum Cyanobacteria in the microbiome of patients with breast cancer on this condition was analyzed (Figure 4). Patients were divided into two groups according to the presence of Cyanobacteria in their microbiome: one without (negative group) and one with Cyanobacteria (positive group). The group without Cyanobacteria had lower-stage cancer. The late-stage (stage III) patients tended to have a higher proportion of Cyanobacteria in their microbiome than the early-stage (stage 0–II) patients. When looking at the ratio of Cyanobacteria according to premenopause or menopause, the ratio of Cyanobacteria in the microbiome of menopausal patients was relatively high. Four types of breast cancer were investigated with the phylum Cyanobacteria level. The hormone-dependent group, luminal A and B patients, accounted for 70.8%, a similar portion of all breast cancer patients. Patients were divided into two groups according to hormone dependence and were compared with the microbiome of phylum Cyanobacteria. These results showed no significant difference in the level of phylum Cyanobacteria in hormone-positive (luminal A and B) and hormone-negative (HER2 and TNBC) breast cancers (Figure S2). Regarding eating habits, the amount of Cyanobacteria in the microbiome of vegetarian patients was the highest. The average age of the patients in the Cyanobacteria-positive group was significantly higher. The BMI (body mass index) and Ki-67 index were similar in the two groups. The ratio of Cyanobacteria was proportional to the tumor size and lymph node metastasis. The presence of lymph node metastasis seemed to be related to a higher amount of Cyanobacteria in the microbiome. Lymph node metastasis might be related to the amount of Cyanobacteria in the microbiome in late-stage patients. The tumor size seemed to be inversely related to the amount of Cyanobacteria in the microbiome; however, this relationship was not statistically significant.

### 3.4. Analysis of the Relationship between the Presence of Cyanobacteria in the Microbiome and Liver Function

**Figure 5:**
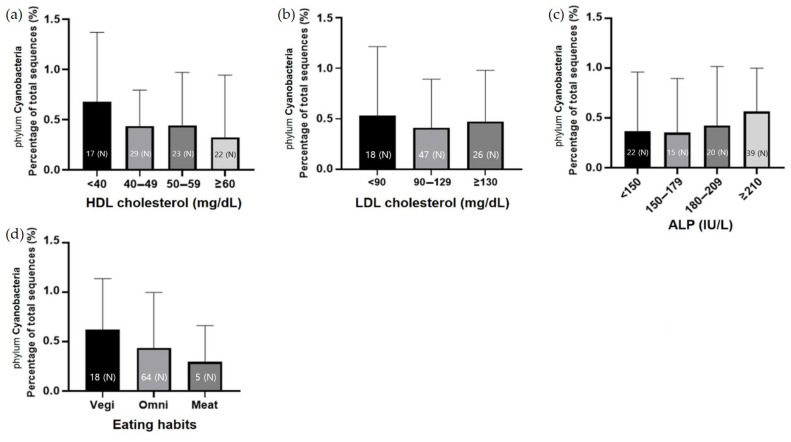
The relationship between the proportion of phylum Cyanobacteria in the microbiome of patients and their serology results or eating habits. The proportion of Cyanobacteria according to (**a**) the level of HDL cholesterol, (**b**) the level of LDL cholesterol, (**c**) the level of ALP, and (**d**) eating habits. LDL cholesterol: low-density lipoprotein cholesterol; HDL cholesterol: high-density lipoprotein cholesterol; ALP: alkaline phosphatase; Vegetarian: patients who eat only vegetarian food; Omnivorous: patients who eat any food well; Meat-based diet: patients who eat meat-based foods daily; (N): number of patients.

**Figure 6:**
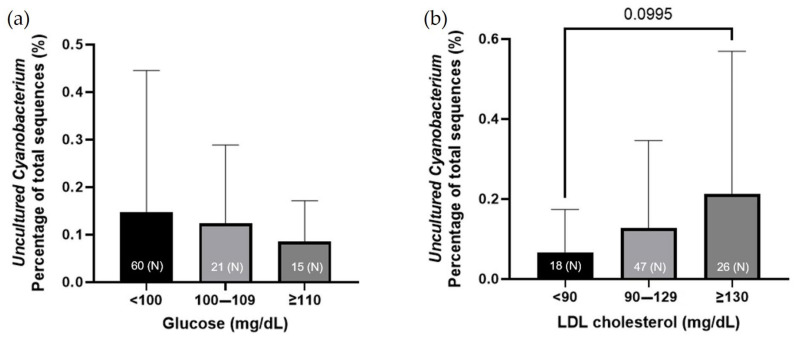
The percentage of *uncultured Cyanobacterium* spp. according to (**a**) the glucose and (**b**) LDL cholesterol levels; *p* = 0.0995. Glucose: fasting glucose level; LDL cholesterol; (N): number of patients.

The presence of phylum Cyanobacteria and the liver function-related serological results were integrated. Serological tests related to liver metabolism and function include the analysis of the levels of glucose, LDL and HDL cholesterol, and ALP. These results were integrated with the abundance of Cyanobacteria in the microbiome and its relevance. The lower the HDL cholesterol level in patients, the higher the percentage of Cyanobacteria in their microbiome. The LDL cholesterol level did not seem to be related to the abundance of Cyanobacteria. Although there was no statistical significance, the higher the ALP level, the higher the percentage of Cyanobacteria in the microbiome of patients. The ratio of Cyanobacteria was high in vegetarians and relatively low in patients who eat meat-based meals every day (Figure 5). The purpose of this study was to integrate the percentage of specific Cyanobacteria species with the serological test results. The proportion of the microbiome assigned to *Uncultured Cyanobacterium* spp. was determined. *Uncultured Cyanobacterium* spp. seem to be related to the LDL cholesterol level rather than to the blood sugar level, and as the ratio of LDL cholesterol increases, the ratio of Cyanobacteria tends to increase (Figure 6).

**Figure 7:**
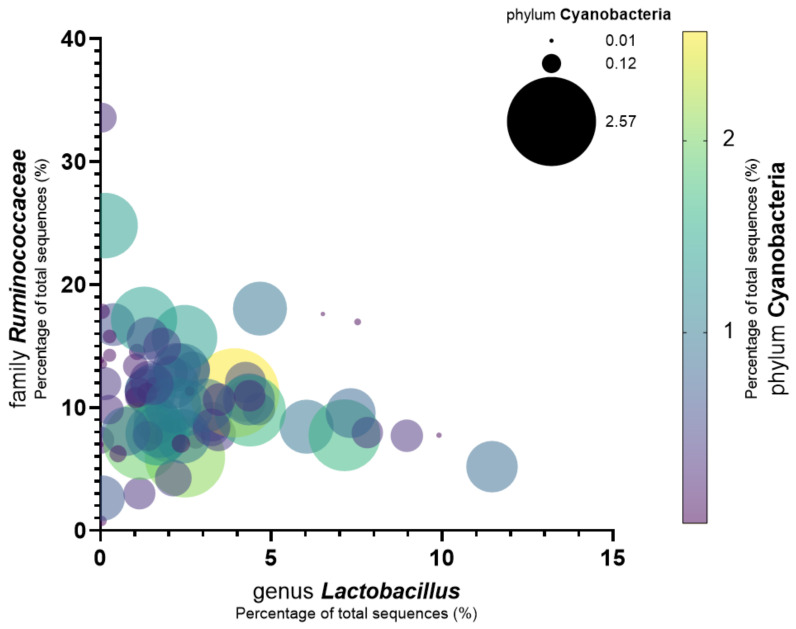
Relationship between the abundance of phylum Cyanobacteria and that of the family *Ruminococcaceae* and the genus *Lactobacillus*, which are associated with liver disease, in the microbiome of patients with breast cancer. As the amount of the family *Ruminococcaceae* increases, the amount of Cyanobacteria decreases. As the amount of the genus *Lactobacillus* increases, the amount of Cyanobacteria also decreases. The bubble size and color correspond to the abundance of Cyanobacteria. There were some values lower than the 0.01 value that was set for the smallest bubble; however, these have been omitted from the graph to ensure clarity.

Since the results of the microbiome analysis in chronic liver disease and cirrhosis patients were investigated in previous studies, the family *Ruminococcaceae* and the genus *Lactobacillus* were known as microbiomes related to liver disease [24,25]. Figure 7 shows the analysis results that confirm the relationship between the abundance of phylum Cyanobacteria and that of other bacteria found in the microbiome that are related to liver disease. The relationship between these three microbiomes was expressed as a bubble plot. The x-axis represents the abundance of the genus *Lactobacillus*, the y-axis represents that of the family *Ruminococcaceae*, and the bubble size and color represent the proportion of Cyanobacteria. The amount of Cyanobacteria was low when the ratio of the family *Ruminococcaceae* and the genus *Lactobacillus* was high, and it was high when the ratio of the family *Ruminococcaceae* and the genus *Lactobacillus* was low. In particular, under a genus *Lactobacillus* abundance of 3.9% or less and an abundance of the family *Ruminococcaceae* of 11.1% or less, a patient group with an abundance of phylum Cyanobacteria of 2% or more was concentrated.

**Figure 8:**
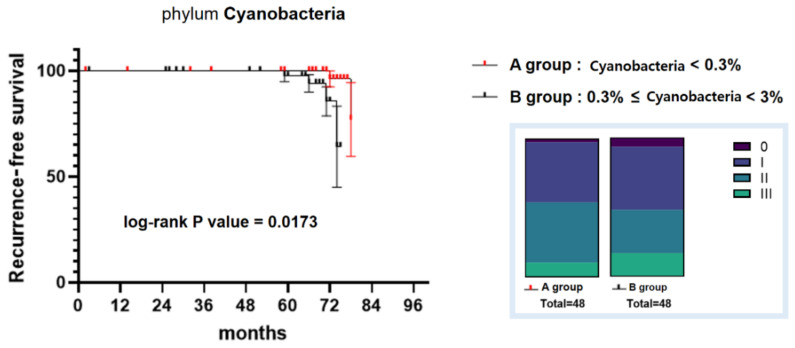
Comparison of the recurrence-free survival between the patients with breast cancer of group A (patients with an abundance of Cyanobacteria < 0.3%) and group B (patients with an abundance of Cyanobacteria ≥ 0.3%). The blue square box on the right shows the stage ratio of the patients of groups A and B when the patients were first recruited. Kaplan–Meier survival analysis was performed, and the log-rank *p* value was 0.0173.

The average percentage of phylum Cyanobacteria found in the microbiome of patients with breast cancer was 0.45%, and it ranged from 0% to less than 3% (Figure 8). Patients were divided into two groups based on a Cyanobacteria abundance value of 0.3%, which is lower than the average value obtained. The group with less than 0.3% of Cyanobacteria was called group A, and the group with more than 0.3% of Cyanobacteria was called group B. When comparing the number of patients by stage in groups A and B, group A has two fewer stage 0 patients, three fewer stage III patients, and five more stage I–II patients than group B. Groups A and B showed differences in the recurrence-free survival. The probability of metastasis and recurrence was significantly lower in group A than in group B. That is, when less than 0.3% of Cyanobacteria was found in the microbiome of patients, the chance of occurrence of metastasis or recurrence was lower.

## 4. Discussion

Cyanobacteria have been around since the beginning of life. Since Cyanobacteria produce oxygen through photosynthesis, they were suitable to live on Earth from the start and were the ones to give rise to other life forms [27]. However, nowadays, Cyanobacteria are considered pollutants that produce cyanobacterial toxins through eutrophication [28]. Above all, cyanobacterial toxins have been pointed out as being harmful to human health [14]. Although the toxins have not been directly investigated in this study, the result of prolonged exposure to Cyanobacteria, which affects their proportion in the human microbiome, was analyzed. To confirm the severity of the effects of Cyanobacteria, cyanobacterial blooms in Republic of Korea were identified using a satellite database. The Ganwol lake, the artificial lake of Cheonsu Bay, is a representative place showing the severity of cyanobacterial blooms. The Okgu reservoir in Gunsan city, a Gaehwa retention reservoir in Buan-gun, and the Yeongju lake in Yeongju city showed severe cyanobacterial blooms. The Geum and Nakdong river estuaries also showed a moderate degree of cyanobacterial blooms. Cyanobacteria flourishment is different depending on the season and changes due to differences in the water temperature and the level of eutrophication. However, the fact that there are places with a level of cyanobacterial contamination this high provides sufficient evidence that the toxicity of Cyanobacteria is likely to influence South Koreans.

The concentration of Cyanobacteria in freshwater is different depending on the environment, changing with the different seasons [29,30]. The degree of exposure to Cyanobacteria was measured as the abundance of phylum Cyanobacteria in the microbiome of patients. We tried to determine the difference between the concentration of Cyanobacteria in the microbiome of patients with breast cancer and the average concentration of Cyanobacteria found in the microbiome of healthy controls, since Cyanobacteria are present in the microbiome of 80% of the population [8]. That is, phylum Cyanobacteria can accumulate in both healthy people and breast cancer patients. Even small changes in the phylum Cyanobacteria concentration in the microbiome can play an important role in human health. In particular, the average abundance of phylum Cyanobacteria in the breast cancer group was 0.45%; however, in the metastatic or recurrent patient group, the average value was 0.76%. Although the difference between these two groups is not statistically significant, this trend suggests the possibility that the presence of Cyanobacteria in the microbiome may influence the occurrence of recurrence or metastasis. Phylum Cyanobacteria were identified in urine as well as serum (Figure S2). It can be expected that the microbiome can affect the whole body, including the breast, through the circulation of body fluids.

The abundance of phylum Cyanobacteria tended to increase with age, which is due to the fact that the amount of Cyanobacteria in the body may accumulate over time. Phylum Cyanobacteria seem to be more abundant in the microbiome of menopausal patients, which seems to be related to the age of the menopausal group patients. Regarding the association with the breast cancer stage, the higher the stage, the higher the abundance of phylum Cyanobacteria. It is likely that metastatic lymph nodes contribute to late-stage cancer due to the abundance of Cyanobacteria in the microbiome. Thus, lymph node metastasis may be associated with the abundance of Cyanobacteria. A correlation between the concentration of phylum Cyanobacteria in the microbiome and several factors related to liver function has been suggested. The lower the HDL cholesterol, the lower the Cyanobacteria abundance in the microbiome. Although this difference was not statistically significant, it suggests that the production of HDL cholesterol is induced when the abundance of Cyanobacteria is low. There was no relationship between the LDL cholesterol and the abundance of Cyanobacteria in the microbiome. However, the higher the LDL cholesterol, the higher the abundance of *Uncultured Cyanobacterium* spp. Although the *p*-value is 0.09, the abundance of *Uncultured Cyanobacterium* spp. in the microbiome tended to affect liver function, such as lipid metabolism. In liver and bone disease, the ALP level increases. The ALP level tends to increase as the abundance of Cyanobacteria increases, which may be correlated with liver function. The main toxin produced by Cyanobacteria is microcystin, which is hepatotoxic. Since the amount of Cyanobacteria in the microbiome is not fatal and accumulates in the body for a lifetime instead of for a short time, no statistical significance was observed. However, there seems to be an association between the presence of phylum Cyanobacteria in the microbiome and liver function and lymph node metastasis in patients with breast cancer.

Chronic liver disease and the human microbiome are closely related according to previous studies [31,32]. Clinical trials in which probiotics and prebiotics are ingested have shown their good effects on the liver enzyme and lipid profile [33]. The abundance of lactic acid bacteria, such as *Lactobacillus*, decreases in chronic liver disease, especially in alcoholic liver disease [24]. The abundance of *Rumonococcoceae* tends to decrease in liver cirrhosis [25]. As the abundance of Cyanobacteria in the microbiome increased, that of these beneficial microorganisms, *Lactobacillus* and *Rumonococcoceae*, decreased. This may be due to the fact that the presence of Cyanobacteria reduces the number of beneficial bacteria in the microbiome and creates a microenvironment that adversely affects the health of the host. The problems associated with Cyanobacteria that have been identified in previous studies are toxin-induced hepatotoxicity and neurotoxicity. The effect of Cyanobacteria on breast cancer prognosis has not been elucidated before. This study is meaningful since it reveals the effect of Cyanobacteria on the metastasis and recurrence in patients with breast cancer. According to the Kaplan–Meier analysis, the prognosis of patients with breast cancer is better when the abundance of Cyanobacteria is lower than 0.3%. This result is statistically significant (log-rank *p* value = 0.0173). By reducing the amount of beneficial bacteria in the microbiome, Cyanobacteria are thought to influence the prognosis of patients with breast cancer.

## 5. Conclusions

Cyanobacteria affect the environment and human health. In this study, the effect of the presence of Cyanobacteria in the microbiome of patients with breast cancer on their condition was investigated. Cyanobacteria are known for their hepatotoxicity and neurotoxicity. In this study, the relationship between the presence of phylum Cyanobacteria in the microbiome of patients with breast cancer and their prognosis was analyzed. A limitation of this study is that certain factors which directly affect the amount of Cyanobacteria in the microbiome of patients, such as the residence place of patients, have not been studied. The specific genus of Cyanobacteria was also not identified. However, this study opens a discussion about the influence of the presence of Cyanobacteria in the surrounding environment on patients with breast cancer and prompts us to rethink the impact of Cyanobacteria-polluted environments on the prognosis of patients with breast cancer.

## Figures and Tables

**Table 1:** Characteristics of the patients with breast cancer.

Characteristics	N (Percentage of the Total Patients) or Concentration
**Female number (N)**	96
**Age (years)**	51.5 ± 11.1
**Body weight (kg)**	58.2 ± 9.4
**Hight (cm)**	158.4 ± 6.6
**BMI (kg/m^2^)**	23.2 ± 3.5
Below 18.5	5 (7.2%)
18.5–24.9	64 (66.6%)
25.0–29.9	21 (21.8%)
30.0–34.9	6 (6.2%)
**Menopause**	
premenopause	48 (50%)
menopause	46 (47.9%)
nonresponse	2 (2.0%)
**Stage**	
0	3 (3.1%)
I	44 (45.8%)
II	36 (37.5%)
III	13 (13.5%)
**Subtype**	
Luminal A	36 (37.5%)
Luminal B	32 (33.3%)
HER2	13 (13%)
TNBC	15 (15.6%)
**Tumor size (cm)**	
<2	53 (55.2%)
2–4.9	38 (39.5%)
≥5	5 (5.2%)
**Lymph node metastasis**	
Negative	65 (67.7%)
Positive	31 (32.2%)
**Serologic tests**	
HDL cholesterol	50.8 ± 13.2 (mg/dL)
LDL cholesterol	113.0 ± 34.3 (mg/dL)
ALP	205.6 ± 70.8 (IU/L)
Glucose	100.1 ± 14.6 (mg/dL)
**Eating habits**	
Omnivorous	64 (66.6%)
Vegetarian	18 (18.7%)
Meat-based diet	5 (5.2%)
Nonresponse	9 (9.3%)

BMI: Body Mass Index; LDL cholesterol: low-density lipoprotein cholesterol; HER2: human epidermal growth factor receptor 2; TNBC: triple-negative breast cancer; HDL cholesterol: high-density lipoprotein cholesterol; ALP: alkaline phosphatase; Glucose: fasting glucose level; Omnivorous: patients who eat any food well; Vegetarian: patients who eat only vegetarian food; Meat-based diet: Patients who eat meat-based foods daily.

## Supplementary Materials

The following supporting information can be downloaded at: https://www.mdpi.com/article/10.3390/jcm11247272/s1, Figure S1: Analysis of Alpha diversity or beta-diversity by dividing the group without phylum Cyanobacteria (Cyanobacteria(−)) and with phylum Cyanobacteria (Cyanobacteria(+)); Figure S2: The level of the phylum Cyanobacteria in patients with breast cancer via serum and urine samples. The relative proportion of phylum Cyanobacteria in serum or urine was compared; Figure S3: The percentage of phylum Cyanobacteria according to breast cancer with hormone-positive (luminal A and B) or hormone-negative (HER2 and TNBC).
