# Supplementary material for: Analysis of the Impact of the Presence of Phylum Cyanobacteria in the Microbiome of Patients with Breast Cancer on Their Prognosis
Source: J Clin Med. 2022 Dec 7;11(24):7272. doi: 10.3390/jcm11247272 (PMC9787457; doi:10.3390/jcm11247272)
Supplement: Supplementary file 1 [file jcm-11-07272-s001.zip › jcm-1921217-supplementary.pdf]

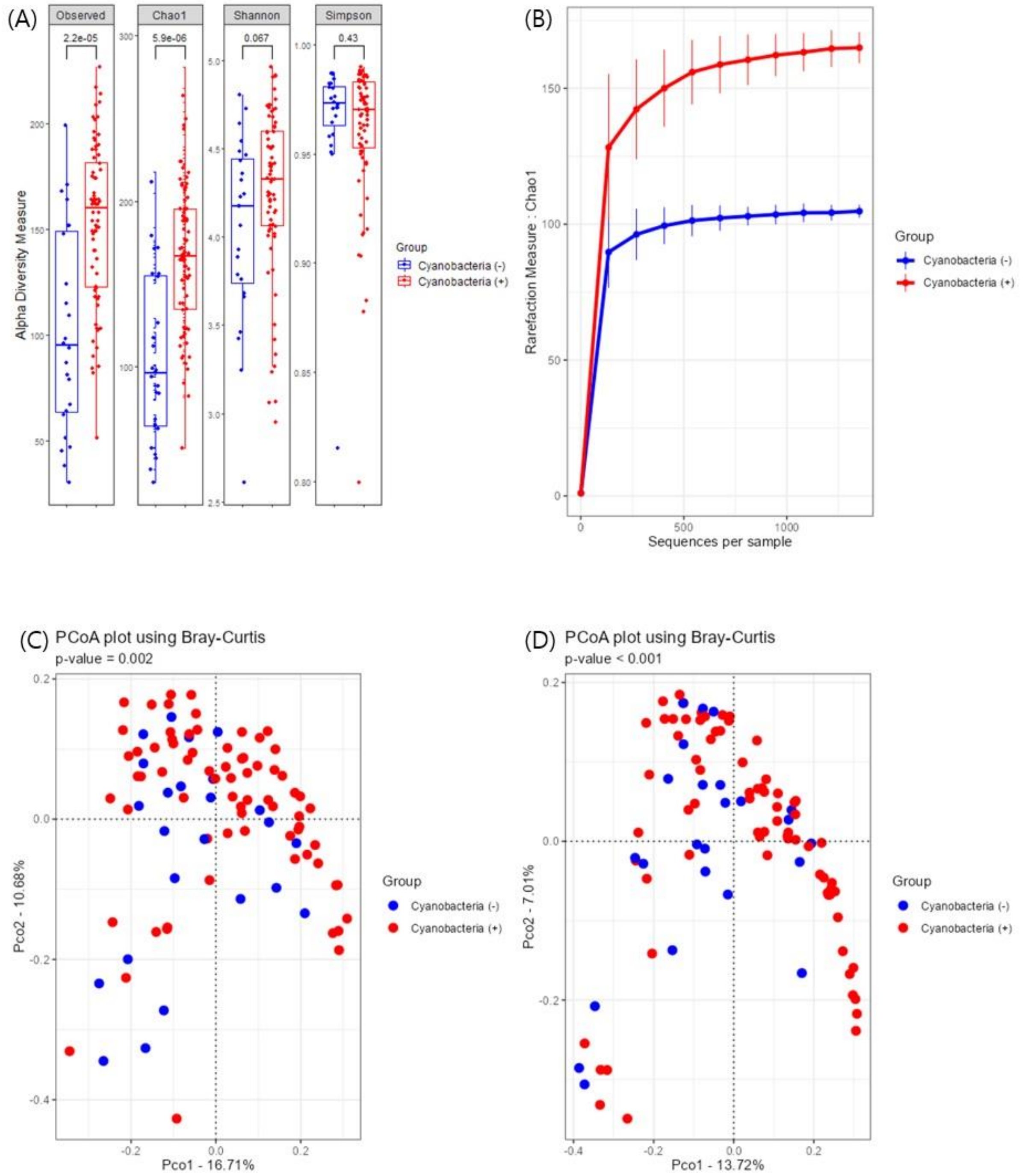

Figure S1. Analysis of Alpha diversity or beta-diversity by dividing the group without phylum Cyanobacteria (Cyanobacteria(-)) and with phylum Cyanobacteria (Cyanobacteria(+)). (A) Comparison of alpha-diversity of the two groups via the observed, Chao1, Shannon, and Simpson methods; (B) Comparison of the Chao1 index of the two groups through box plot; (C) Comparison of beta-diversity of the two groups at the genus level through PCoA. (D) Comparison of beta-diversity of the two groups at the species level through principal coordinates analysis (PCoA).

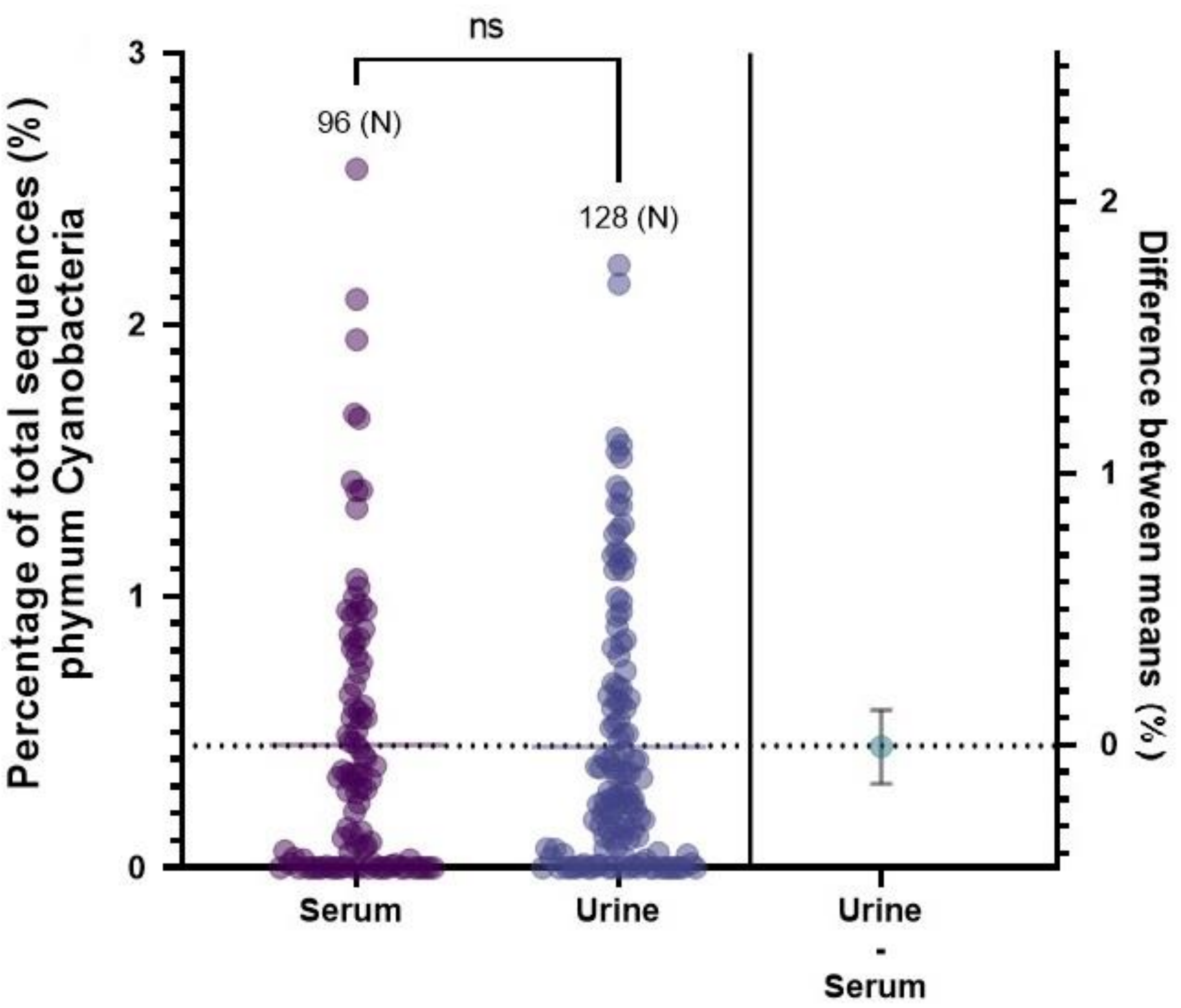

Figure S2. The level of the phylum Cyanobacteria in patients with breast cancer via serum and urine samples. The relative proportion of phylum Cyanobacteria in serum or urine was compared; Urine-Serum: Mean difference in phylum Cyanobacteria between the two sample groups.

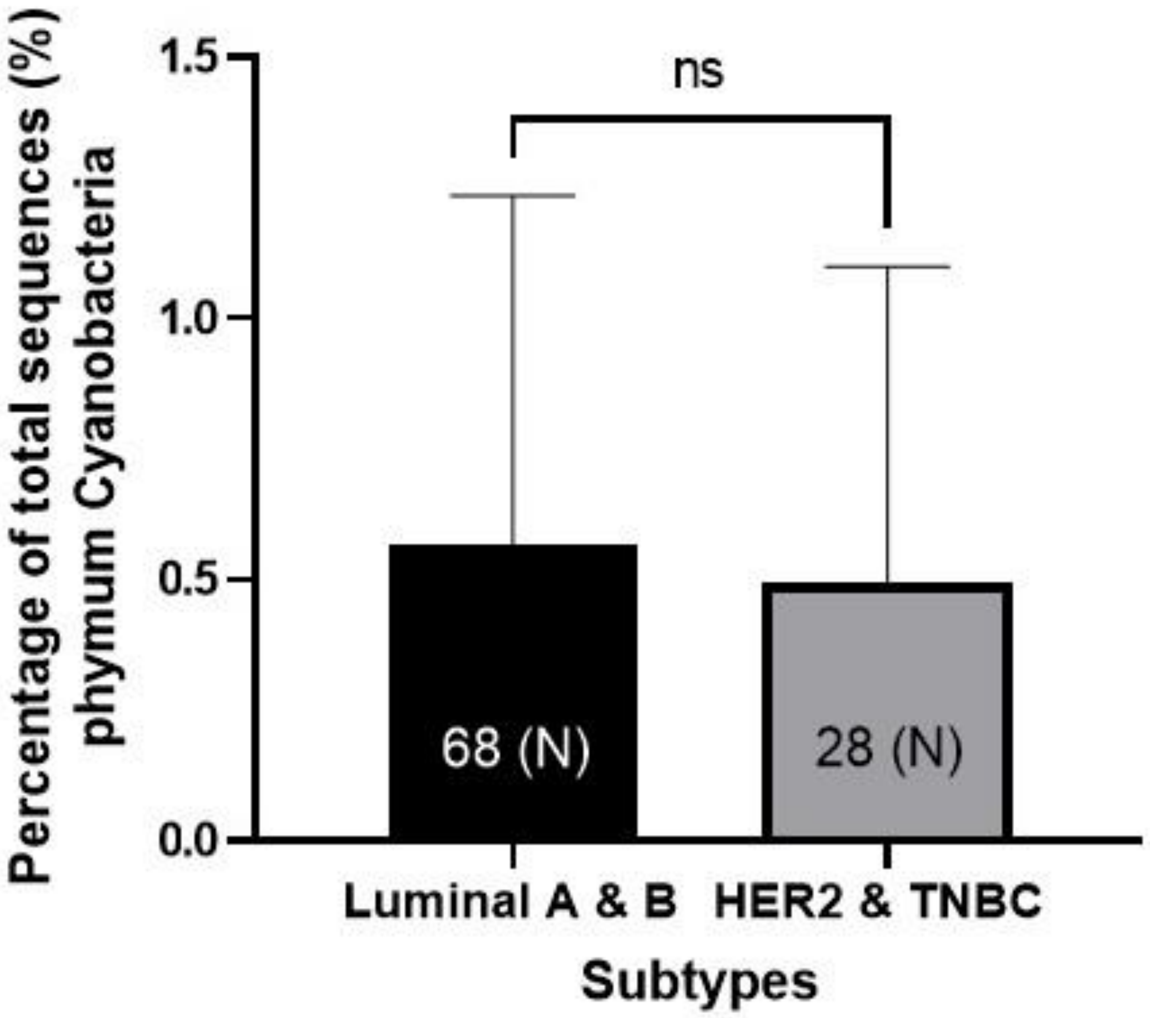

Figure S3. The percentage of phylum Cyanobacteria according to breast cancer with hormone-positive (luminal A and B) or hormone-negative (HER2 and TNBC); HER2: human epidermal growth factor receptor 2; TNBC: triple-negative breast cancer; (N): numbers of patients; ns: no significance.
